# Paracoccidioimycosis and white individuals: Susceptibility and biogeographic aspects in an important endemic area in Brazil

**DOI:** 10.1371/journal.pntd.0009086

**Published:** 2021-02-09

**Authors:** Luciana Bonome Zeminian de Oliveira, Amanda Manoel Della Coletta, Taiane Priscila Gardizani, Ligia Vizeu Barrozo, Hélio Amante Miot, Julio De Faveri, Luciane Alarcão Dias-Melicio

**Affiliations:** 1 São Paulo State University (UNESP), Medical School of Botucatu, Laboratory of Immunopathology and Infectious Agents—LIAI, UNIPEX—Experimental Research Unity, Sector 5, Botucatu, São Paulo State, Brazil; 2 University of São Paulo (USP), Department of Geography, School of Philosophy, Literature and Human Sciences, São Paulo, São Paulo State, Brazil; 3 São Paulo State University (UNESP), Medical School of Botucatu, Division of Dermatology and Radiotherapy, Botucatu, São Paulo State, Brazil; 4 São Paulo State University (UNESP), Medical School of Botucatu, Department of Pathology, Botucatu/SP–Brazil; University of Tennessee, UNITED STATES

## Abstract

Paracoccidioidomycosis (PCM) is a neglected mycosis most commonly occurring in Latin America. The etiologic agents are thermo dimorphic fungi of the genus *Paracoccidioides*, and cause an important granulomatous response in affected tissues. The Botucatu Medical School, from São Paulo State University (UNESP), is a PCM study pole, located in São Paulo State Midwest region, which is classified as a hyperendemic area in the Southeast region in Brazil. This study aimed to perform a retrospective epidemiological, geographical, and clinical analysis by the information available in medical records. It was listed as socio-demographic data along with clinical characteristics from patients diagnosed and treated during a 10-year period in Botucatu, totaling 177 patients with Paracoccidioidomycosis confirmed by the histopathological test. It was observed that the main clinical presentation was the chronic type (76,3%), most commonly identified in white male individuals over the age of 29 years old, smokers, and alcoholics, providing evidences for the first time that white individuals were more affected by the disease, in comparison to non-white individuals that may be more resistant to infection. This data opens new avenues for study within ancestry, resistance and susceptibility in paracoccidioidomycosis.

## Author summary

Paracoccidioidomycosis (PCM) is an important neglected tropical disease that affects many countries in Latin America, including Brazil. The main affected population is rural workers who suffer from an important debilitating disease, besides facing massive economic damage in their lives. Although many studies evaluated the socio-demographic data of patients with PCM and identified many white individuals affected, this is the first study that has managed to compare the data of patients treated in a leading study of the disease with regional population data provided by the Brazilian Institute of Geography and Statistics (IBGE), with regards to ethnicity. The difference between white individuals over non-white individuals found in this study was significant, suggesting a susceptibility of white individuals to PCM. However, the ethnic background is self-reported; therefore, a genetic ancestry investigation could allow other correlations concerning ancestry markers, susceptibility, and resistance to infection.

## Introduction

Paracoccidioidomycosis (PCM) is a disease most prevalent in South American countries such as Brazil, Colombia, Venezuela, and Argentina. This illness is characterized as a systemic granulomatous mycosis that affects mainly the lungs but can disseminate to other organs by hematogenous lymph circulation resulting in several different clinical manifestations [1].

The etiologic agents of PCM are the fungi of the *Paracoccidioides* genus. They are thermal dimorphic microorganisms that grown as yeast *in vivo*, in host tissues or *in vitro* cultures at 37°C in enriched culture media. The fungi are also found as mycelium at room temperature ranging from 4 to 28°C [1].

It was believed that *Paracoccidioides brasiliensis* was the sole etiologic agent causing PCM, but studies of genomics and phylogeny pointed out that there is more than one species able to cause the mycosis. In 2006, it was described three cryptic species (S1, PS2, and PS3) and in 2009, *Paracoccidioides lutzii* [2,3]. Later, it was shown a fourth cryptic species, PS4 [4]. Recently, a new nomenclature for these species was suggested as follows: *P*. *brasiliensis* (for S1), *P*. *americana* (PS2), *P*. *restrepiensis* (PS3), and *P*. *venezuelensis* (PS4) [5]. These species are not uniformly distributed in Latin America, and some are more prominent in determined regions than in others [2,4,6–13].

PCM commonly occurs in male rural workers with 30–50 years old in constant contact with vegetation and soil. It is an illness that has a severe socioeconomic impact since it affects the leading providers impairing their ability to perform their gainful activity [13]. This mycosis is also considered a neglected disease, emphasizing a vicious circle of poverty and poor health care conditions in the different countries [14,15].

The soil is the most likely habitat of the *Paracoccidioides* spp., as suggested by isolates in Argentina, Venezuela, Colombia, and Brazil [16]. The fungus lives in a saprobic form in the soil as mycelium confirmed by isolation and molecular tests [16–18]. Some domestic and wild animals have been provided as valuable epidemiological markers to reveal their physical location [19].

The infectious form (conidia) are inhaled and then travel to the lungs where they are deposited. In the lungs, they go through a transformation to yeast form caused by the increase of temperature (36°-37°C); that way, the yeast form can establish the disease [1].

PCM can manifest in different forms, and the severity of the disease depends on gender, age, and conditions of the host immune system and the amount of inhaled conidia. It can be asymptomatic, characterizing the sub-clinical infection or the progressive disease that could either be acute/subacute or chronic forms [1].

The primary infection usually is lacking in clinical manifestations; therefore, the mycosis can present itself with the two different patterns already described. The acute/subacute form (juvenile type) is commonly observed in children, teenagers, and adults under 30 years old, which represents less than 10% of the cases. Contrarily from the chronic type, the lungs are generally not involved, and clinically, the case evolves rapidly, and the patient’s condition can be severely hindered in a few weeks or months [1].

The chronic type affects typically male rural workers, as mentioned above, and symptoms progress slowly; thus, patients take longer to seek medical care. It can affect a single organ or system, or it can be disseminated to multiple organs through hematogenous lymph circulation [1].

Great efforts have been made towards understanding the biology of *Paracoccidioides* spp. and the mechanisms of disease and host immune response. Due to the high incidence and importance of PCM in large regions of Latin America, it is essential to understand better the pathology and epidemiologic aspects of the disease, which will reflect on the improvement of quality of life, treatment, and diagnosis for patients [20].

Therefore, analysis of a data collection of a hyperendemic area can help clarify and raise essential questions about PCM, creating new insights to be explored. Thus, our study brings new and relevant results related to the susceptibility of white male individuals to PCM, opening new perspectives for the evaluation of the ancestry of the affected individuals.

## Materials and methods

### Ethics statement

This research was conducted after approval of the Research Ethics Committee of UNESP Medical School of Botucatu (CAAE: 31053514.1.0000.5411). The Research Ethics Committee exempted the use of consent forms for our patients given the retrospective nature of our study.

### Study setting

It was enrolled 177 patients with confirmed PCM by a histological analysis performed by the Department of Pathology from UNESP Medical School of Botucatu in a period of 2004 to 2014. Their medical records were collected and evaluated, with the socio-demographic profile and clinical history of all the patients enrolled. In this study, those who used alcohol and cigarettes daily were classified as alcoholics and smokers.

Secondly, a comparison was performed between the Brazilian Institute of Geography and Statistics (IBGE) data [21], published from 2004 to 2015, demonstrating the composition of the rural worker population in our area with the data of our patients surveyed in this study.

### Data and statistical analysis

For the maps demonstrating the naturality and provenance of patients, the software ArcGIS v.10.1 for georeferencing (Esri) was utilized, and for the area calculation, the Google maps platform was used.

For the statistical analysis of variables like gender, ethnicity, smoking, and alcohol intake, taking into consideration the correlation between the groups, the Fisher exact test using the BioStat 5.3 software and Pearson’s chi-square test goodness of fit and residual analysis of contingency table was used. The analysis of the patient’s age was done by descriptive analysis using the GraphPad Prism 8.0.0. Software.

Finally, the clinical and demographic variables were assessed through an exploratory multiple correspondence analysis model [22].

## Results

### Analysis of the area of PCM cases

**Fig 1:**
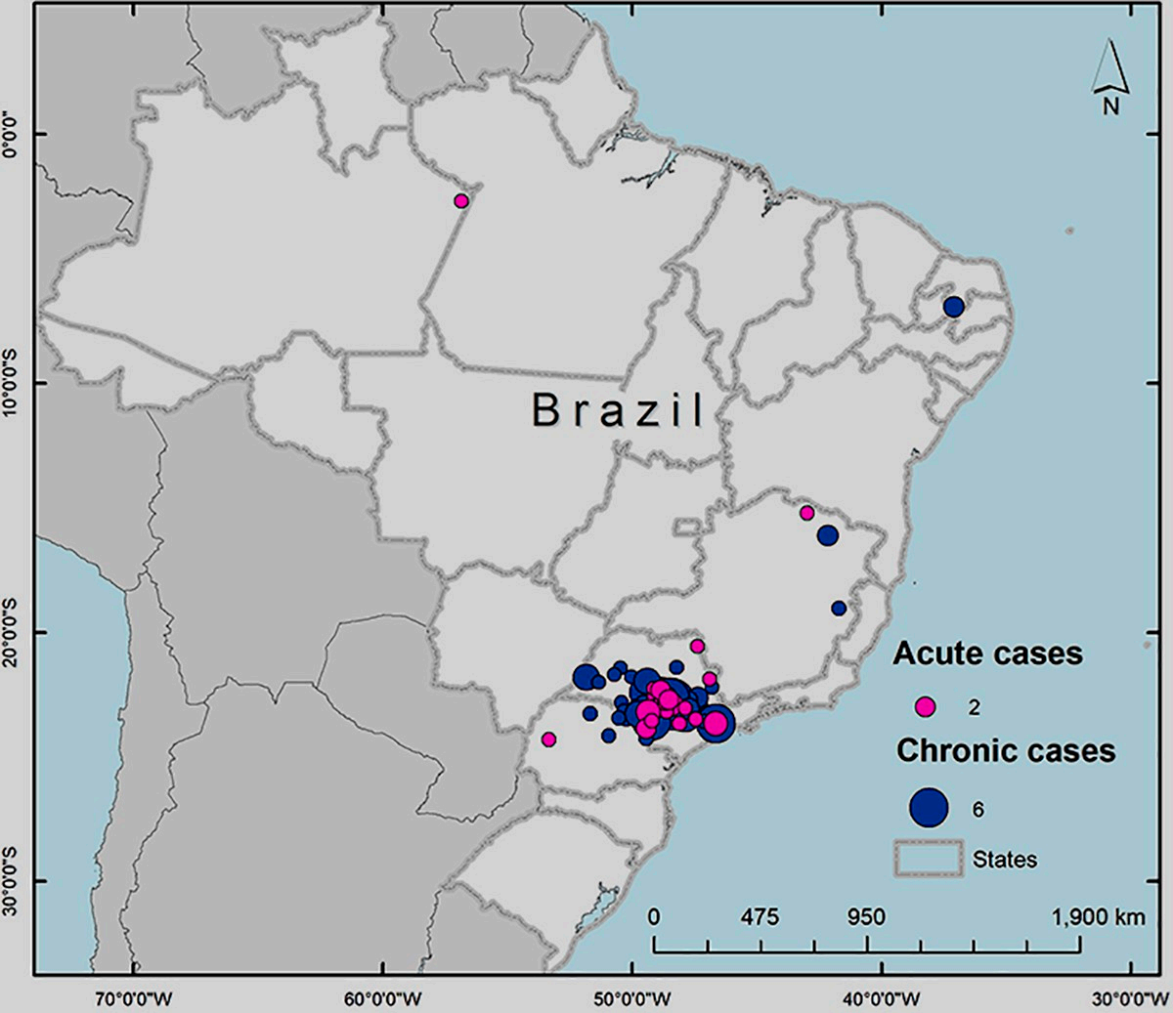
Map demonstrating the distribution of the 177 patients birthplace. This map was constructed using the software ArcGIS v.10.1 for georeferencing (Esri).

It was identified that most of our patients are residents from the São Paulo State with a few cases from Paraná and the Minas Gerais States and two patients from the north and northeast regions (Amazonas and Paraiba States), as demonstrated by pink (acute form) and blue (chronic form) markers in Fig 1.

As already mentioned, the higher concentration of patients was born in São Paulo State, covering an area of 91.312 square feet according to Google maps platform analyses. Paraná State is the second largest area of patient’s place of origin, covering an area of 27.657 square feet.

**Fig 2:**
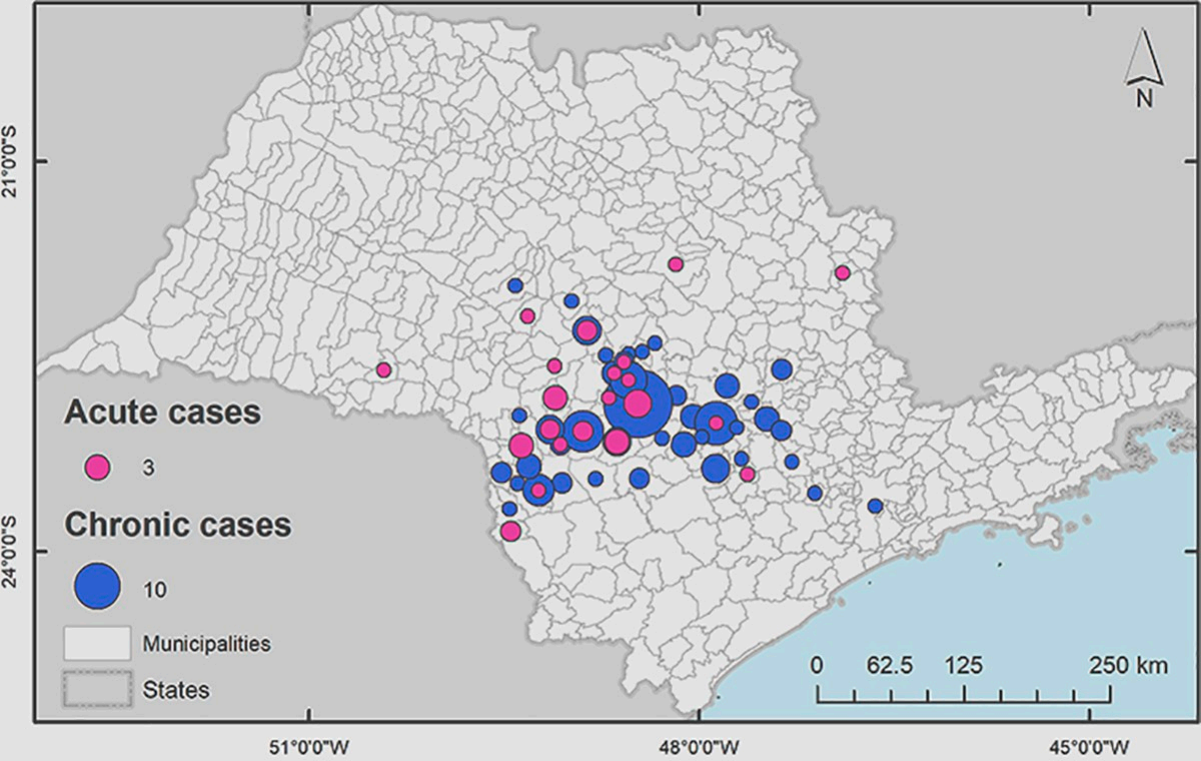
Concentration of patients in the Botucatu region at the moment of the PCM diagnosis and treatment beginning. Map demonstrating the concentration of patients during treatment in the Botucatu region. This map was constructed using the software ArcGIS v.10.1 for georeferencing (Esri).

When it was analyzed the area where the patients lived, the place where they came from at the moment of the PCM diagnosis and treatment beginning, the group was predominantly concentrated in municipalities at São Paulo State central west region, around the city of Botucatu, in a 47.772 square feet area, as shown in Fig 2 and Table 1.

**Table 1:** São Paulo State’s cities where patients with PCM were currently living at the time of first attendance at the Institution, according to the clinical form presented (2004–2014).

City of Residence During Treatment	Clinical Form N (%)			City of Residence During Treatment	Clinical Form N (%)		
	Chronic	Acute/ Subacute	Total		Chronic	Acute/ Subacute	Total
Agudos	1 (0,7)	1 (3)	2	Laranjal Paulista	9 (6,6)	1 (3)	10
Angatuba	2 (1,5)		2	Limeira	2 (1,5)		2
Anhembi	1 (0,7)		1	Macatuba	1 (0,7)		1
Arandu	2 (1,5)	1 (3)	3	Mineiros do Tietê	1 (0,7)		1
Araraquara		1 (3)	1	Óleo	1 (0,7)		1
Arealva	1 (0,7)		1	Paranapanema	1 (0,7)		1
Areiópolis	3 (2,2)	1 (3)	4	Pederneiras	4 (2,9)	2 (5)	6
Assis		1 (3)	1	Pereiras	1 (0,7)		1
Avaí		1 (3)	1	Piracicaba	3 (2,2)		3
Avaré	8 (5,8)	2 (5)	10	Pirajuí	1 (0,7)		1
Barra Bonita	1 (0,7)		1	Piraju	3 (2,2)	3 (8)	6
Bofete	1 (0,7)		1	Pirambóia	1 (0,7)		1
Boituva	1 (0,7)		1	Porangaba	3 (2,2)		3
Botucatu	23 (16,8)	4 (10)	27	Pratânea	1 (0,7)	1 (3)	2
Capivari	3 (2,2)		3	Rio das Pedras	1 (0,7)		1
Cerqueira César	4 (2,9)	2 (5)	6	Riversul		2 (5)	2
Conchas	3 (2,2)		3	São Manuel	7 (5,1)	1 (3)	8
Dois Córregos	1 (0,7)		1	São Paulo	1 (0,7)		1
Elias Fausto	2 (1,5)		2	São Roque	1 (0,7)		1
Fartura	2 (1,5)		2	Taguaí	1 (0,7)		1
Iaras		3 (8)	3	Taquarituba	5 (3,6)	1 (3)	6
Igaraçu do Tietê	2 (1,5)	1 (3)	3	Tatuí	4 (2,9)		4
Iperó		1 (3)	1	Tejupá	3 (2,2)		3
Itaí	2 (1,5)		2	Tietê	1 (0,7)		1
Itaporanga	1 (0,7)		1	Vargem Grande do Sul		1 (3)	1
Itatinga	4 (2,9)	3 (8)	7	Not Declared	12 (8,8)	6 (15)	18
Itu	1 (0,7)		1				

### Analysis of patient socio-demographic and clinical data

From the 177 patients with confirmed PCM, were done a profile resulting from the analysis of their socio-demographic and clinical data.

The clinical characteristics of our patients demonstrating the PCM profile in the Botucatu region are shown in Table 2. Our patients showed that the chronic type of PCM is more common in our region, predominating in 76.3% of the cases. Also, the disease is more frequently presented in males (80.75%), white individuals (91.9%), smokers (76.3%) (Table 2), with superior age to 50 years old, since of the 135 patients with the chronic form, 96 patients were over 50 years old (71.1%). In the acute type, the data is more uniform between genders, although also predominant in white individuals (83.4%), non-smokers (66.76%) and non-alcoholic (80.95%) (Table 2).

**Table 2:** Characteristics of PCM patients treated in Botucatu from 2004 through 2014.

Characteristics		Acute (n = 42)	Chronic (n = 135)	p-value
Patient’s age (years)*		37,6 (16–61)	60,48 (29–98)	-
Gender**	Male	54,76% (23/42)	80,74% (109/135)	<0,001
	Female	45,24% (19/42)	19,26% (26/135)	<0,001
Ethnicity**	White	83,33% (35/42)	91,85% (124/135)	NS (>0,05)
	Non-white	16,67% (7/42)	8,15% (11/135)	NS (>0,05)
Smokers**	Smoker	33,33% (14/42)	76,30% (103/135)	<0,0001
	Non-smoker	66,67% (28/42)	23,70% (32/135)	<0,0001
Alcoholics**	Alcoholic	19,05% (8/42)	54,07% (73/135)	<0,0001
	Non-alcoholic	80,95% (34/42)	45,93% (62/135)	<0,0001

* Values expressed as mean(min-max).

** Values expressed as percentage.

p-value indicate the differences between acute and chronic form.

NS—not significant–no difference between groups (p>0,05).

For the statistical analysis of variables like gender, ethnicity, smoking, and alcohol intake, the Fisher Exact Test was done, using the BioStat 5.3 software.

### Comparison of ethnicity data with IBGE data

According to data given by the Brazilian Institute of Geography and Statistics (IBGE) [21], published from 2004 to 2015, demonstrating the composition of the rural worker population in our area, it was possible to perform a statistical analysis comparing our data with that expected for their natural region. It was performed three scenarios of comparison between IBGE data and the collection of patients assessed using Pearson’s chi-square test (p<0.001) after 10.000 (bootstrap) simulations (Table 3).

**Table 3:** Comparison between Botucatu’s patient data and IBGE rural worker population data.

PCM Clinical Form	Ethnicity	PCM patients	IBGE Data	p-value
**Total**	White	159	373.171	<0,001
	Non-White	18	55.887	
**Acute form**	White	33	373.171	0,825
	Non-White	7	55.887	
**Chronic form**	White	126	373.171	<0,001
	Non-White	11	55.887	

Comparison using Pearson’s chi-square test goodness of fit.

Bootstrap 10.000 simulations.

First, it was compared the ethnicity of the patients with the natural composition of São Paulo State rural worker population. This analysis expresses a higher proportion of white individuals (84.3%-93.2%) among the PCM affected population when confronted with rural workers population of the São Paulo State, according to IBGE (p<0.001) (Table 3).

The second analysis was between the acute cases of PCM occurring in white and non-white individuals with the natural composition of São Paulo rural population showing a slightly high proportion of white patients (70.0%-92.5%) among the PCM affected population (p = 0.825) (Table 3).

The last analysis was performed between the chronic cases of PCM occurring in white and non-white individuals compared with the natural composition of São Paulo rural population, demonstrating a vastly higher proportion of white patients (86.1%-94.9%) among the PCM affected population (p<0.001) when compared with the total rural community (Table 3).

IBGE also provided data from the year 2015, showing the natural composition of the rural worker population post-2014. It was identified a decrease of 33% in the total rural population of São Paulo State in that period, with a reduction of the white population and a slight increase in the non-white population. This new population design must give us more possibilities to better understand these findings in the near future.

### Multiple correspondence analysis

**Fig 3:**
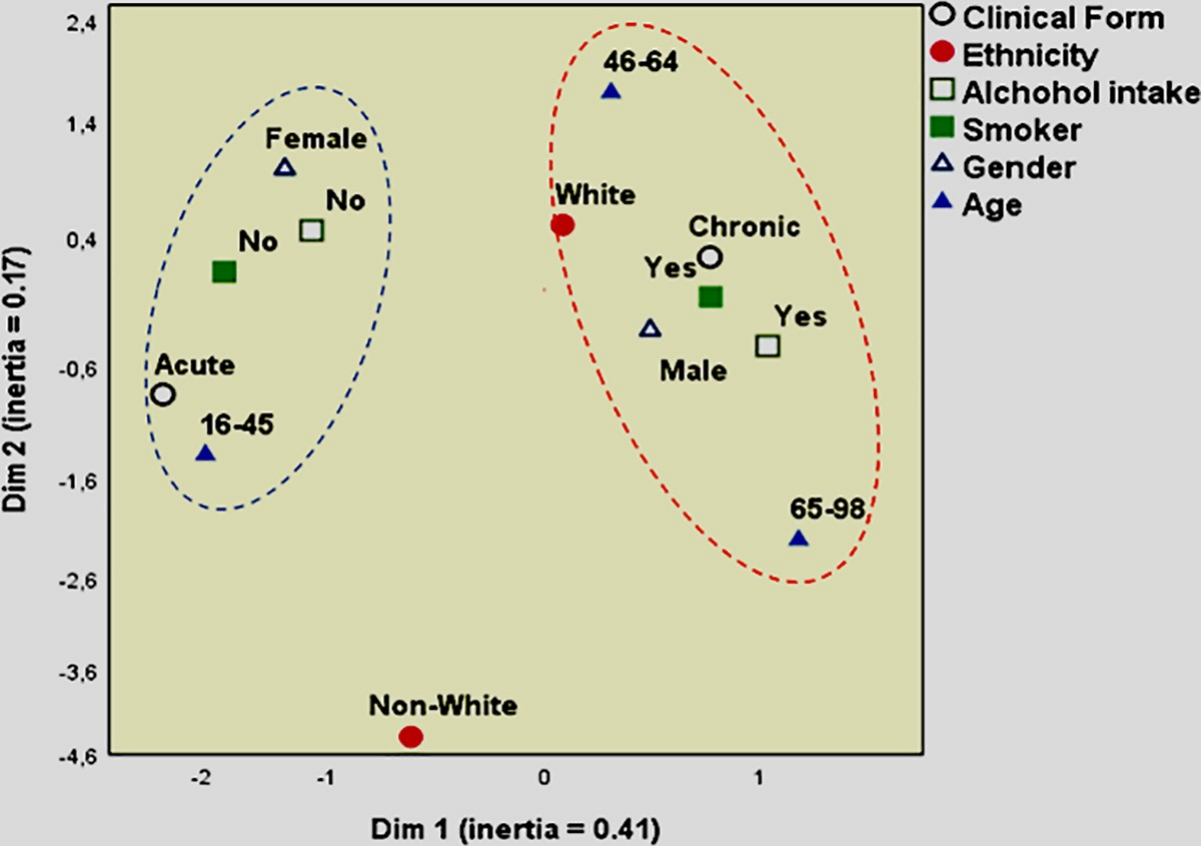
Perceptual map from multiple correspondence analysis for variables of PCM epidemiological markers.

Also, it was performed a multiple correspondence analysis model to assess the variables and try to understand the scenario of PCM epidemiological markers in a multivariable way, which is shown in Fig 3. This analysis demonstrated that when epidemiological markers were analyzed all together, the acute clinical form was correlated with non-smokers, non-alcohol intake, females, and age between 15–45 years old (Fig 3). The chronic form was correlated with smoking, alcohol intake, males, and age between 46–98 years old (Fig 3). The non-white individuals were far in the map and did not correlate with any of the markers (Fig 3), corroborating our results demonstrated above.

## Discussion

Even though PCM is considered a neglected tropical disease and does not have a global impact when compared to other infections, a meticulous data collection of the pathobiology could give us a better understanding of it and possibly improve treatments as well as prevention measures.

PCM has a higher incidence in rural workers that has intense contact with soil [23]. The incidence rate between males and females shows that it preferentially affects male individuals of mixed race [1,24]. It is most prevalent among males because one of the hormonal factors (estrogen) confers protection to the women hindering the transformation to the yeast form in the body, that it is the pathogenic form [1,24].

Our socio-demographic data collection shows that some of these markers corroborate with the known literature regarding PCM, such as clinical forms, gender, smoking, and alcoholism. Our data also show that PCM occurs in patients of all ages, including kids and teenagers with the acute form type, and is more predominant in males more than 30 years old, that presents the chronic form, as already described [1,14,25].

However, one fact that caught our attention was the difference in the incidence between white and non-white individuals affected by the disease. Some studies identified higher numbers of white subjects affected but could not detect differences when compared to the typical ethnic composition of the area that reflected the local population [26,27]. Until now, no study identified a racial predisposition to the development of PCM [28,29], although it was already seen in a miscegenated population of Southern Brazil in which blacks and mulattoes show more disseminated lesions of the acute/subacute form than white patients [27–29]. There are also studies discussing the role of melanin produced by the pathogen that could confer a higher virulence to the fungi [30], but nothing about why non-whites are less prone to this infection.

Bellissimo-Rodrigues *et al*. [27] showed a higher incidence of white individuals affected with both clinical forms of the disease that were attended in the Hospital of Clinics of the School of Medicine of Ribeirão Preto of the University of São Paulo. However, an association of PCM and race background, for both men and women, in the first two decades of life, shows that all patients presented the acute/subacute form of the disease, independent of their sex and ethnicity. In the subsequent decades of life, they identified that the disease was more frequent in non-white individuals, according to the study. It was suggested that it could be due to the ethnic composition of the area population [27].

In 2011, Belissimo-Rodrigues *et al*. [26] identified that 79% of patients were white individuals, while 8,2% were black individuals, and 12,8% were mulatto. The authors discussed that ethnicity does not seem associated with PCM, once the distribution of patients was comparable with the population of the area of Ribeirão Preto regarding skin color [26].

In fact, according to the Brazilian Institute of Geography and Statistics (IBGE) [21], from 2004 to 2015, the rural worker population in the State of São Paulo, that is the main population affected by the disease, was composed by 70,6% of white and 29,4% of non-white individuals, and even though, our analysis detected a statistical difference between affected white and non-whites individuals when compared with the rural population of the area. These data could indicate a resistance of the non-white population against the fungus, once Brazil has achieved nearly universal access to health-care services for the population, especially in the State of São Paulo, with public access to the Unified Health System (Sistema Único de Saúde—SUS) [31]. Thus, it is unlikely that the white individuals are more likely to seek medical diagnosis and treatment than non-white individuals, especially in relation to the characteristics of the disease that has severe symptoms, with severe organ involvement, causing the patient debilitation, and that will require medical care.

Studies have been shown that African American people were more likely than white ones to carry allelic variants that increase the expression of the pro-inflammatory cytokines, such as IL-1Α, IL-1Β, IL-6, TNF-α, and IL-18, and less likely to carry allelic variants known to increase expression of IL-10, which could account to their resistance profile to infections [32]. Studies have shown an association of polymorphisms in immune response genes with genomic ancestry [33,34].

Nédélec *et al*. [35] evaluated the transcriptional response of macrophages to live bacterial pathogens and identified the effects of African versus European ancestry on it, showing an ancestry-associated difference in the gene regulatory response to infection. The study demonstrated that African ancestry specifically predicts a more robust inflammatory response and reduced intracellular bacterial growth.

Yao *et al*. [36] identified strong ancestral footprints in inflammatory chemokine regulation in women, showing that CCL11 and CCL2 were strongly associated with a percent of African ancestry, as well as higher levels of IL1RA and IFNα2. Another study searching to identify differences in innate cytokine responses between European and African children showed differences in TLR responsiveness in whole blood, with an enhanced pro-inflammatory response to TLR2/1, TLR2/6, and TLR4 stimulation for African children [37]. Studies evaluating serum cytokine levels between African and European Americans showed that these levels varied by race and could contribute to lung cancer differently between these two populations [38]. A study taking account of a southeastern Brazilian population in São Paulo State showed an estimation of European ancestry (63%) and African ancestry (22%) [39]. It was showed that the mean proportions of European ancestry differed between the genotypes of the TLR6 (P249S) gene and in the TLR1 (I602S) gene, being 602S allele, that is more present in European ancestry, related to a hypo-responsiveness [39].

Besides, another important fact that corroborates our results is that the causes of death, according to race in the São Paulo State, demonstrated that white individuals died more from infectious diseases, neoplasms, and other diseases than the non-white ones [40].

Furthermore, in our multiple correspondence analysis model, it was shown that the acute form had more proximity to non-smoking, no alcohol intake females between the ages 15–45 years old; while the chronic form aligned to smoking and alcohol intake males, between the ages of 46–98 years old. The non-white individuals did not correlate with any of the markers, which also supports our data. More profound research into the reasons for this high incidence among races could be immensely clarifying.

Also, based on the Brazilian Institute of Geography and Statistics data, a new census was performed during the year of 2015 and showed an accentuated decrease in the rural workers in the State of São Paulo. This new data could have a significant impact on epidemiology and must be followed to better understand these factors on paracoccidioidmoycosis epidemiology in the future.

However, given the nature of self-declaration of the ethnicity of our data, it could open a high possibility for future research to clarify the questions raised by our data collection analysis. Genetic studies could provide useful information for the investigation of the disease, especially in cases with a difference of proportion in ethnic groups. Given the high miscegenation of the Brazilian population, an assessment of ancestry using informative ancestry markers (AIMs) could exempt the subjectivity of self-declared ethnicity and reported family origin [32].

Even with our data corroborating the known characteristics of the infection, the questions raised in this study, about white individuals being more affected by the disease than non-white ones, suggest that non-white individuals are more resistant to infection. It could inspire different studies, such as the genetic ancestry of the patients affected by this disease correlating with immune response.

These results are part of the Luciana Bonome Zeminian de Oliveira´s doctoral thesis concluded at Medical School of Botucatu, São Paulo State University (UNESP) [41].
